# Labyrinth Stimulation

**Published:** 1919-01

**Authors:** 


**Labyrinth Stimulation.** By H. B. Lemere. *Journal A. M. A.*, Sept. 14, 1918.

The author suggests that in the study of the application of the physiology and function of the semicircular canals, in the present interest aroused by the aviation tests, there is one method of approach that has not been used, and yet offers a promising field for investigation. That is a more careful study of the actual anatomic position of these canals and their relations to the movements of the head on the body, together with the conjugate movements of the eye. To simplify matters, he says, this subject should be considered mainly in the erect position of the body. He goes over the anatomy of the canals and the mechanics of the motions of the eyes in their relation to each other, and finds that there is a direct relationship between stimulation of the following canals and the action of the following muscles: the superior canals and the superior and inferior recti; the horizontal canals and the internal and external recti, and the inferior canals and the obliques. Also, the horizontal canals are stimulated by the movements of the head nearly in a horizontal plane, the superior in a longitudinal plane, and the inferior in a transverse plane. The erroneous conception of the positions of those canals should be corrected and they should be called horizontal, longitudinal aud transverse, respectively.

